# Robotic‐assisted restricted kinematic alignment in total knee arthroplasty: A multicenter retrospective assessment of coronal phenotypes and early postoperative outcomes

**DOI:** 10.1002/jeo2.70646

**Published:** 2026-01-19

**Authors:** Ravi Teja Rudraraju, Sanjay Bhalchandra Londhe, Ponnanna Karineravanda Machaiah, Supreet Bajwa, Kunal Aneja, Police Jayaram Reddy, Kanakanala Jangi Reddy, Dolly Singh

**Affiliations:** ^1^ Department of Orthopaedics Apollo Hospitals Hyderabad Telangana India; ^2^ Department of Orthopaedics SVS Medical College Mahbubnagar Telangana India; ^3^ Department of Orthopaedics Criticare Asia Hospital Andheri, Mumbai Maharashtra India; ^4^ Department of Orthopaedics Sparsh Hospital Yeshwanthpur, Bengaluru Karnataka India; ^5^ Department of Orthopaedics, Wockhardt Hospitals Mumbai Central Mumbai Maharashtra India; ^6^ Department of Orthopaedics, Shalimar Bagh Max Super Speciality Hospital Shalimar Bagh, New Delhi Delhi India; ^7^ Managing Director Naveda Healthcare Centers New Delhi Delhi India; ^8^ Department of Clinico Marketing and Medical Writing Meril Healthcare Pvt. Ltd. Vapi Gujarat India

**Keywords:** Coronal Plane Alignment of the Knee (CPAK), functional outcomes, phenotype‐based alignment, restricted kinematic alignment, robotic‐assisted total knee arthroplasty

## Abstract

**Purpose:**

The Coronal Plane Alignment of the Knee (CPAK) classification enables phenotype‐based total knee arthroplasty (TKA), but its application in robotic‐assisted TKA (RATKA) remains underexplored. We aim to describe the distribution of CPAK phenotypes in patients undergoing RATKA using a restricted kinematic alignment (rKA) protocol, evaluate alignment correction within rKA boundaries and report early postoperative functional outcomes. We hypothesized that RATKA would achieve alignment targets within the rKA range while demonstrating expected postoperative functional recovery.

**Methods:**

This multicenter retrospective study included 200 patients with primary knee osteoarthritis between June and November 2024 at five high‐volume centres. All procedures followed a rKA protocol, with target correction within ±3° of constitutional alignment. CPAK distribution, demographic variations, achieved alignment and early postoperative functional outcomes measured by the Knee Injury and Osteoarthritis Outcome Score (KOOS) and Forgotten Joint Score (FJS) were analysed.

**Results:**

The cohort (mean age 62.9 years; 60.5% female) showed preoperative arithmetic hip–knee–ankle angle of −5.8° ± 4.6° and joint line obliquity of 173.2° ± 4.1°, predominantly varus (71.5% Type I). Type I alignment was more frequent in males (73.4%), females (70.2%) and those aged >61 years. Of the 71.5% CPAK Type I, 63.5% remained Type I with correction within 0° ± 3°, while 8% were corrected to Type II. All achieved alignment within rKA limits (mean postoperative hip–knee–ankle 173.0° ± 3.2°). Mean postoperative lateral distal femoral angle and medial proximal tibial angle were 85.1° and 87.7°, respectively. Significant improvements were observed in KOOS (80.9 ± 2.3 vs. 40.6 ± 3.5, *p* < 0.05) and FJS (75.9 ± 1.4 vs. 49.8 ± 1.3, *p* < 0.05) at 6 months. No early revisions occurred.

**Conclusion:**

RATKA performed with a rKA strategy, achieved postoperative alignment within planned rKA limits across CPAK phenotypes. Early functional outcomes at 6 months were satisfactory and consistent with expected postoperative recovery following TKA; however, in the absence of a comparator group, these improvements cannot be attributed solely to the robotic platform or alignment strategy.

**Level of Evidence:**

Level II, retrospective study.

**Abbreviations**
aHKA: arithmetic hip–knee–ankle angle
CPAK: Coronal Plane Alignment of the Knee
CT: computed tomography
FJS: Forgotten Joint Score
HKA: hip–knee–ankle
iKA: inverse kinematic alignment
JLO: joint line obliquity
KA: kinematic alignment
KOOS: Knee injury and Osteoarthritis Outcome Score
LDFA: lateral distal femoral angle
MA: mechanical alignment
MPTA: medial proximal tibial angle
OA: osteoarthritis
OTS: Optical Tracking Sensor
PS: posterior stabilized
RATKA: robotic‐assisted total knee arthroplasty
rKA: restricted kinematic alignment
ROM: range of motion
SPSS: Statistical Package for the Social Sciences
TKA: total knee arthroplasty

## INTRODUCTION

Advances in orthopaedic research have facilitated increasingly precise evaluation of knee alignment and morphology [23]. The Coronal Plane Alignment of the Knee (CPAK) classification system serves as a pivotal tool for characterizing coronal plane deviations [16]. Mechanical alignment (MA) has historically been the dominant strategy in total knee arthroplasty (TKA), supported by long‐term clinical survivorship data [21]. The goal of MA is to provide a level joint line and a balanced mechanical axis, which has long been believed to offer an ideal mechanical environment for the long‐term functionality of knee prosthesis [21]. However, MA does not account for substantial variability in native coronal alignment across individuals, which may alter knee biomechanics when corrected uniformly [4].

Alternative alignment philosophies, including MA, kinematic alignment (KA), inverse kinematic alignment (iKA), restricted kinematic alignment (rKA) and functional alignment (FA), have been introduced to account for native anatomical variation [5]. These approaches aim to restore constitutional joint geometry and ligament balance to better reflect native knee mechanics and motion [7, 10, 12, 18, 27]. However, there have been recurrent inquiries concerning the appropriate degree of alignment of coronal components, which have triggered ongoing discussions regarding their long‐term resilience [18].

While various alignment philosophies seek to respect constitutional limb alignment, a major challenge has been the absence of a standardized method to describe native coronal morphology across populations. The CPAK classification proposed by MacDessi et al. provides this structure by combining arithmetic hip–knee–ankle angle (aHKA) representing constitutional limb alignment and joint line obliquity (JLO) indicating joint line orientation to categorize knees into nine phenotypes [13, 14]. CPAK has since been validated in radiologic and operative contexts, including recent work demonstrating reproducibility under rKA frameworks [4] and convergence with other alignment concepts [23]. The CPAK framework, therefore, enables phenotype‐informed planning and allows surgeons to contextualize alignment targets relative to native constitutional parameters rather than universal neutral correction. Furthermore, surgeons need to identify and understand these differences in knee characteristics among populations to effectively plan and provide individualized arthroplasty treatment. This information is particularly relevant in the Indian context, where genetic and lifestyle factors may influence coronal knee characteristics. Prior studies often used inconsistent classification systems, limiting direct comparisons between datasets [16, 21].

Robotic‐assisted TKA (RATKA) has emerged as a key technological development within the last decade, enabling accurate execution of patient‐specific alignment plans with reduced variability [20]. Contemporary work has shown that robotics can support functional and personalized alignment strategies, improve gap balance and deliver precise coronal and sagittal component positioning [7, 25]. Recent reports also highlight the capacity of robotic systems to implement nuanced alignment philosophies such as rKA, allowing surgeons to target narrow alignment windows while minimizing unintended deviations [7, 17]. Despite these advances, limited evidence exists from India on how robotic platforms perform when executing rKA across diverse CPAK phenotypes, particularly in a multicenter setting.

This study, therefore, aimed to describe the distribution of CPAK phenotypes in Indian patients undergoing RATKA using an rKA protocol, assess postoperative alignment relative to rKA boundaries across these phenotypes and report early postoperative outcomes. The hypothesis was that RATKA performed with an rKA strategy would achieve postoperative alignment within rKA limits across CPAK phenotypes while demonstrating early postoperative recovery expected following RATKA.

## METHODS

### Study design

This retrospective observational cohort study was conducted between June and November 2024 at five high‐volume arthroplasty centres. This study adheres to STROBE guidelines for observational studies in its design and reporting. Consecutive patients were identified through electronic hospital records and surgical logs, based on those who had undergone RATKA using the MISSO robotic system (Meril Healthcare Pvt. Ltd. Vapi, India) during the study period. As the study relied solely on previously collected clinical and computed tomography (CT)/radiographic data without direct patient contact or interventions, ethical committee approval was not required, in accordance with institutional guidelines. However, all procedures followed the ethical guidelines set by the institutional research committee in compliance with the Declaration of Helsinki.

### Inclusion and exclusion criteria

**Figure 1:**
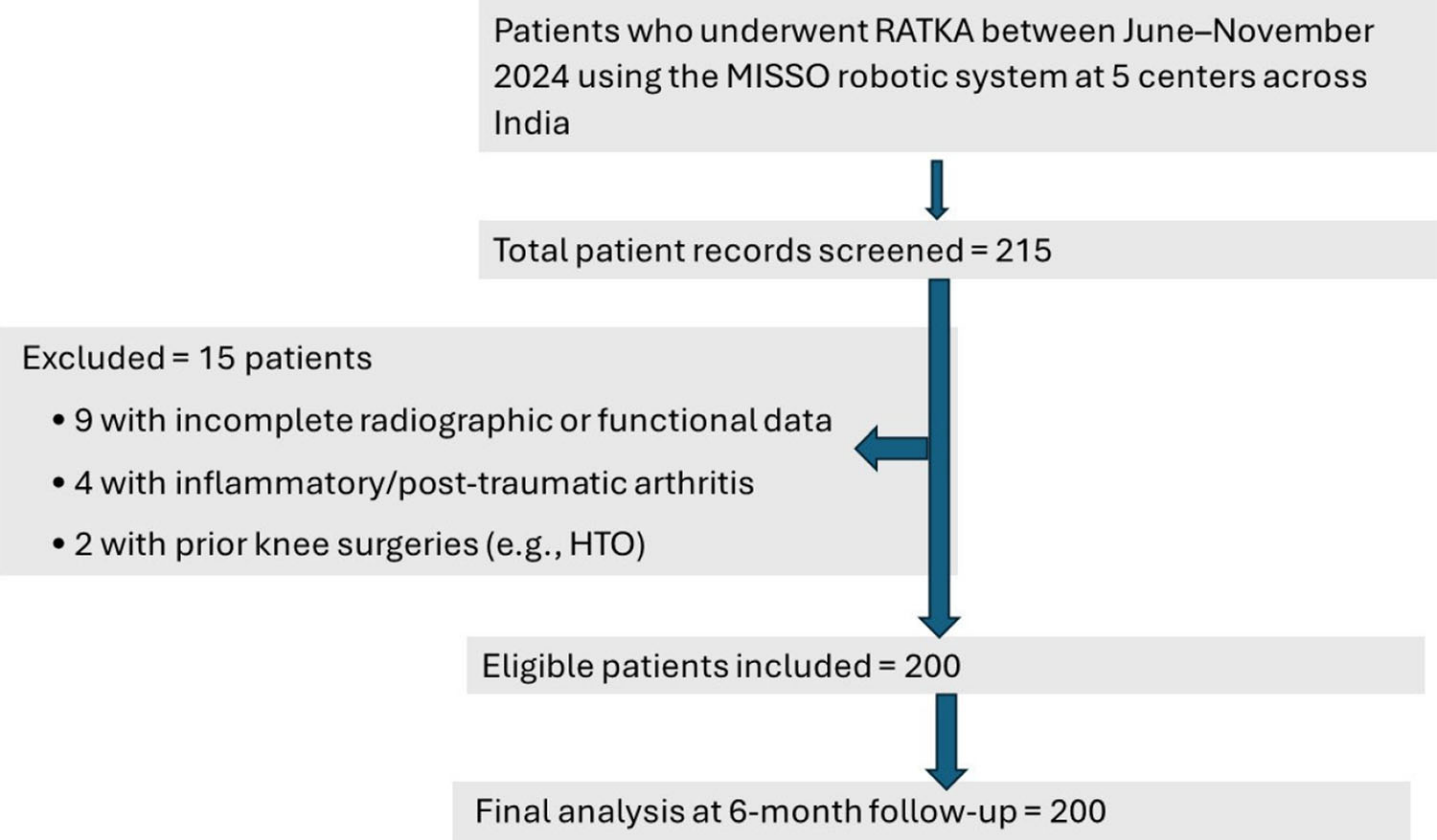
Flow diagram depicting patient selection. A total of 200 patients met the inclusion criteria and were included in the final analysis. HTO, high tibial osteotomy; RATKA, robotic‐assisted total knee arthroplasty.

Key inclusion criteria were end‐stage knee osteoarthritis (OA) indicated for TKA (Kellgren–Lawrence Grade III and IV) and availability of pre‐ and postoperative full‐length standing radiographs. We excluded data of the patients with significant extra‐articular deformity, those with inflammatory arthritis or secondary post‐traumatic arthritis and any requiring complex implants (hinged prostheses) or additional osteotomies (Figure 1). In addition, patients with incomplete CT scans or those who declined RATKA were not included. Preoperative CT scans were conducted on both knees of the study population. No patients were excluded based on outcome or implant‐related complications to ensure data integrity and reduce reporting bias.

### Sample size calculation

The required sample size was estimated to achieve 90% power for detecting a clinically meaningful paired difference of 1.5 points in functional outcome scores (Knee injury and Osteoarthritis Outcome Score [KOOS] and Forgotten Joint Score [FJS]), assuming a standard deviation of 5.0 for paired differences. This distribution was selected based on typical patient‐reported outcome measure (PROM) variability reported in the literature for early postoperative TKA outcomes [8]. Under these assumptions, a minimum of 119 patients were required. The enroled sample of 200 patients exceeded this threshold, supporting adequate statistical power for the study's primary functional endpoints.

### The MISSO robotic system

The MISSO system is a fully active robotic platform used for TKA, enabling surgeon‐defined planning and execution of bone resections under robotic control. The system incorporates preoperative three‐dimensional planning based on CT data, allowing surgeons to define target alignment and joint‐line orientation consistent with rKA parameters. Bone resections are carried out robotically within predefined boundaries, helping maintain alignment within the planned limits [15].

### Surgical technique: rKA approach

All procedures were planned and executed according to a rKA protocol, with the goal of reproducing each patient's constitutional alignment and joint line orientation within ±3° of the native aHKA and joint line orientation, rather than correcting to a neutral axis. This approach sought to maintain each patient's CPAK‐defined phenotype while respecting safe alignment limits to avoid excessive JLO or extreme coronal deviation. Surgical planning was based on individual anatomy, and component placement was restricted within ±3° of the targeted values [26].

Femoral and tibial bone cuts were customized using preoperative templating and soft tissue releases were minimized. Registration was performed after removal of obstructing osteophytes to capture native ligamentous tension before proceeding with resections. Ligament balancing was primarily achieved through bone resections and component positioning. Femoral component was positioned to replicate the patient's native distal femoral joint line within 0° ± 3° of the distal femoral angle in the coronal plane. Femoral rotation was aligned with either the posterior condylar axis or the surgical transepicondylar axis based on intraoperative anatomy. Tibial component was oriented to restore the native proximal tibial JLO, within 0° ± 3° of aHKA. The posterior tibial slope was matched to the native anatomy, typically within the range of 3°–7°. Intraoperative verification included real‐time dynamic assessment of medial and lateral gaps in both extension and flexion. Final implant positioning and restoration of JLO were confirmed using robotic assistance, ensuring precise alignment, joint line symmetry and soft tissue balance in accordance with the targeted CPAK phenotype.

### Data analysis

The continuous variables were expressed as means ± standard deviation. The proportions of knees identified according to the CPAK classification are visually shown as scatter plots. Subgroup analyses were performed by sex, age group and geographic distribution (North vs. South India, based on the location of participating centres). Preoperative CT scans were used to calculate aHKA, lateral distal femoral angle (LDFA) and medial proximal tibial angle (MPTA). Angles were measured by two independent raters (orthopaedic surgeons experienced in radiographic assessment), and inter‐rater reliability was evaluated using the intraclass correlation coefficient (ICC). Postoperative alignment was assessed using long‐leg radiographs at 6 months. The relationship between alignment changes (aHKA, JLO) and improvements in functional outcomes (KOOS, FJS) was expressed using the coefficient of determination (*R* ^2^) to indicate the strength of association. The data were compiled using Microsoft Excel (2016) and analysed using SPSS Version 22.

## RESULTS

Table 1 provides a summary of the baseline features of the study population. The average age of the patients was 62.9 ± 7.7 years. Female population (*n* = 121, 60.5%) was dominant among the study patients. All patients were implanted with Freedom posterior stabilized (PS) knees (MAXX Orthopedics, Inc.).

**Table 1:** Baseline demographic and knee phenotypes of the study patients.

Demographic parameters	Mean ± SD		Range (min–max)
Age, years	62.88 ± 7.69		40–83
Gender, *n* (%)			
Male	79 (39.50%)		
Female	121 (60.50%)		
Body mass index (kg/m^2^)	29.6 ± 4.3		20–42
Stage of osteoarthritis	I	10 (5%)	
	II	95 (47.5%)	
	III	57 (28.5%)	
	IV	38 (19%)	

Abbreviation: SD, standard deviation.

### Knee phenotype

Preoperatively, the average aHKA of the participants was −5.8° ± 4.6°, suggesting a range of native alignment between neutral (0° ± 2°) and constitutional varus (aHKA ≤ 2°). The average obliquity of the joint line in the cohort was 173.2° ± 4.1°, suggesting that the highest point of the joint line is located near apex distal orientation in the vast majority of the population, except for two patients classified as Type IX.

**Figure 2:**
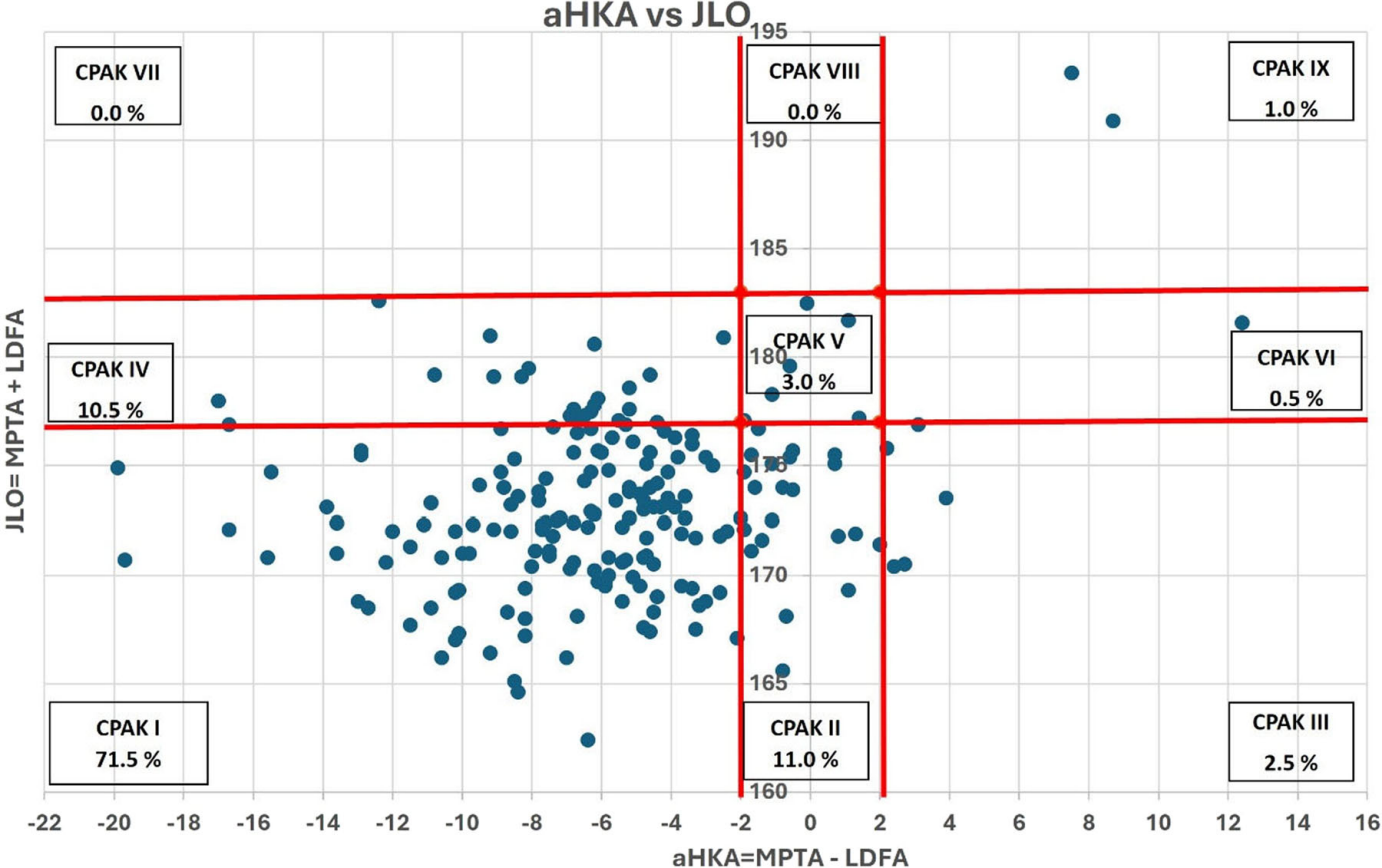
Distribution of CPAK type in the study population. aHKA, arithmetic hip–knee–ankle angle; CPAK, Coronal Plane Alignment of the Knee; JLO, joint line obliquity; LDFA, lateral distal femoral angle; MPTA, medial proximal tibial angle.

The study population was categorized preoperatively into the nine potential phenotypes, according to the boundaries set by the CPAK classification. The majority of the knees (*n* = 143, 71.5%) exhibited the CPAK Type I phenotype, while a smaller proportion (*n* = 22 knees, 11%) had the CPAK Type II phenotype. The other distribution order based on frequency was as follows: Type IV (*n* = 21, 10.5%), Type V (*n* = 6 knees, 3%) and Type III (*n* = 5 knees, 2.5%), while Type VI (*n* = 1, 0.5%) and Type IX (*n* = 2, 1%) were rare. No patients exhibited Type VII or Type VIII (Figure 2, Table 2).

**Table 2:** Distribution of knee phenotypes of the study patients.

CPAK type	Total	Male	Female	Phenotype
Type 1 (aHKA: less than minus 2 and JLO less than 177)	143 (71.5%)	58 (40.56%)	85 (59.44%)	Varus knees/apex distal
Type 2 (aHKA 0+/−2 and JLO less than 177)	22 (11%)	4 (18.18%)	18 (81.82%)	
Type 3 (aHKA greater than 2 and JLO less than 177)	5 (2.5%)	2 (33.33%)	3 (66.67%)	
Type 4 (aHKA less than minus 2 and JLO between 177 and 183)	21 (10%)	11 (55%)	10 (45%)	Neutral knees
Type 5 (aHKA 0 + /−2 and JLO between 177 and 183)	6 (3%)	2 (33.33%)	4 (66.67%)	
Type 6 (aHKA greater than 2 and JLO between 177 and 183)	1 (0.5%)	1 (100%)	0	
Type 7 (aHKA less than minus 2 and JLO greater than 183)	0	0	0	Valgus knees/apex proximal
Type 8 (aHKA 0+/−2 and JLO greater than 183)	0	0	0	
Type 9 (aHKA greater than 2 and JLO greater than 183)	2 (1%)	0	2 (100%)	

Abbreviations: aHKA, arithmetic hip–knee–ankle angle; CPAK, Coronal Plane Alignment of the Knee; JLO, joint line obliquity.

### Sub‐analyses

**Figure 3:**
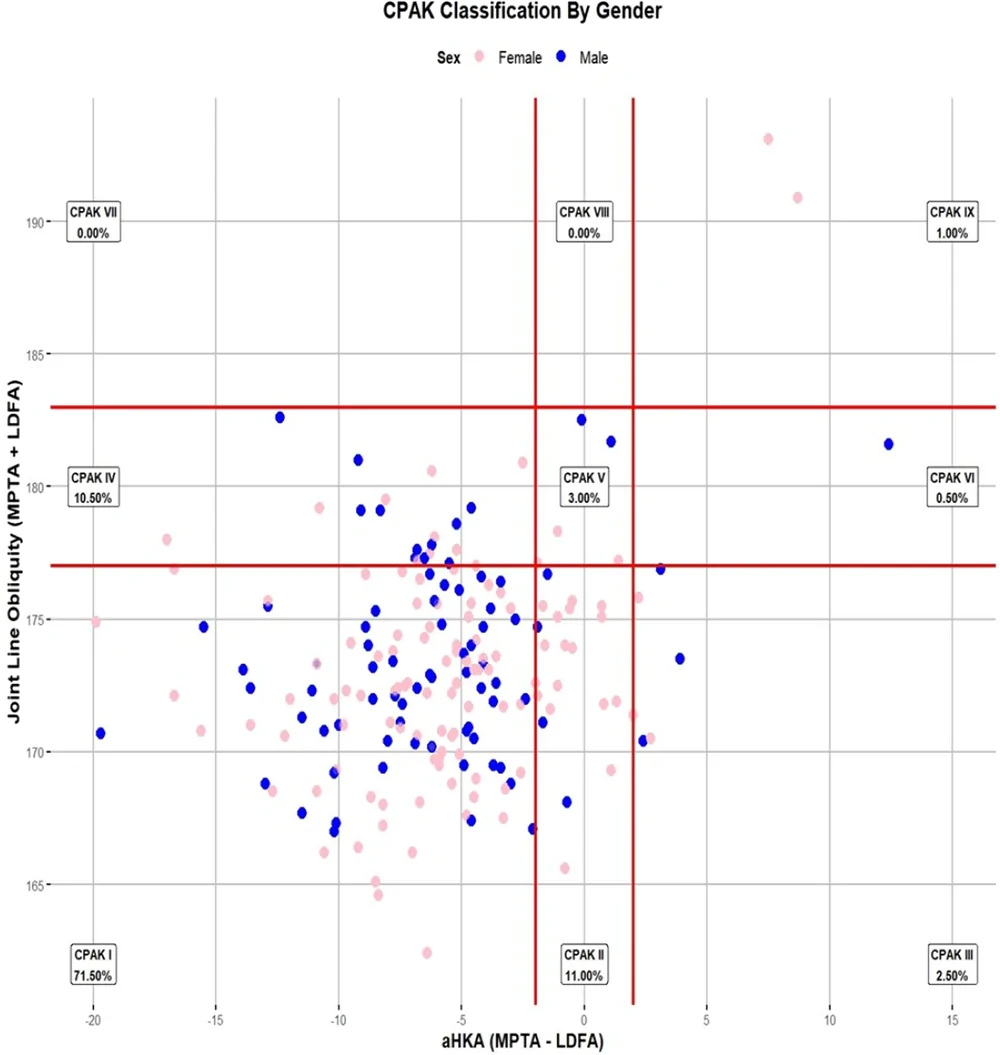
CPAK classification by sex. Scatter plot of arithmetic hip–knee–ankle angle (aHKA = MPTA − LDFA; *x*‐axis, degrees) versus joint‐line obliquity (JLO = MPTA + LDFA; *y*‐axis, degrees) for the study cohort (*n* = 200). Pink points represent female knees and blue points represent male knees. Red grid lines delineate CPAK boundaries; labels indicate CPAK types and the proportion of the cohort in each category. Negative aHKA values denote varus alignment, and higher JLO values indicate a more distal‐apex joint line. Percentages within callouts show the overall cohort distribution. CPAK, Coronal Plane Alignment of the Knee; JLO, joint line obliquity; LDFA, lateral distal femoral angle; MPTA, medial proximal tibial angle.

An analysis based on gender revealed that there were a total of 79 males, accounting for 39.5% of the population and 121 females, making up 60.5% of the population. Out of the 79 men, the majority of them were classified as CPAK Type I (*n* = 58, 73.4%), followed by CPAK Type IV (*n* = 11, 13.9%) and CPAK Type II (*n* = 4, 5.1%) (Figure 3). Among women, the majority exhibited CPAK Type I alignment (*n* = 85, 70.2%), followed by CPAK Type II (*n* = 18, 14.9%) and CPAK Type IV (*n* = 9, 7.4%).

**Figure 4:**
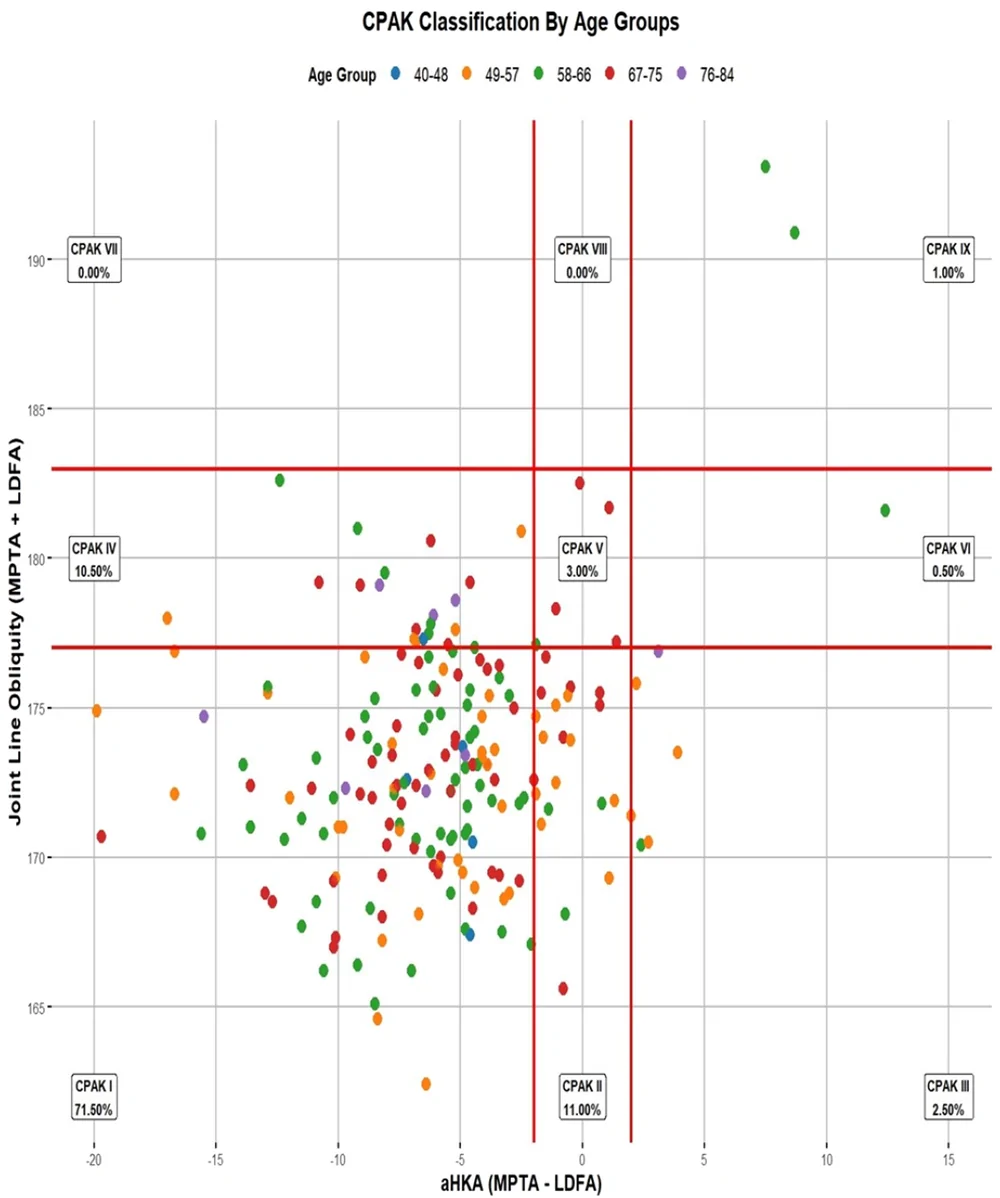
CPAK classification by age group. Scatter plot of aHKA (*x*‐axis, degrees) versus JLO (*y*‐axis, degrees). Red grid lines mark CPAK boundaries with type labels and cohort percentages. Negative aHKA indicates varus alignment. This figure illustrates the spread of CPAK phenotypes across age strata within the same cohort. aHKA, arithmetic hip–knee–ankle angle; CPAK, Coronal Plane Alignment of the Knee; JLO, joint line obliquity; LDFA, lateral distal femoral angle; MPTA, medial proximal tibial angle.

Using age analysis, we categorized patients into two groups: Group 1 consisted of patients aged between 40 and 60 years (*n* = 75), while Group 2 included patients aged 61 years and above (*n* = 125). The classification of CPAK depending on age group is depicted in Figure 4. In Group 1, 51 patients exhibited Type I CPAK classification, while in Group 2, a total of 92 patients showed Type I apex‐distal varus knee alignment. The rarest form, known as CPAK IX, was identified in two female patients.

**Figure 5:**
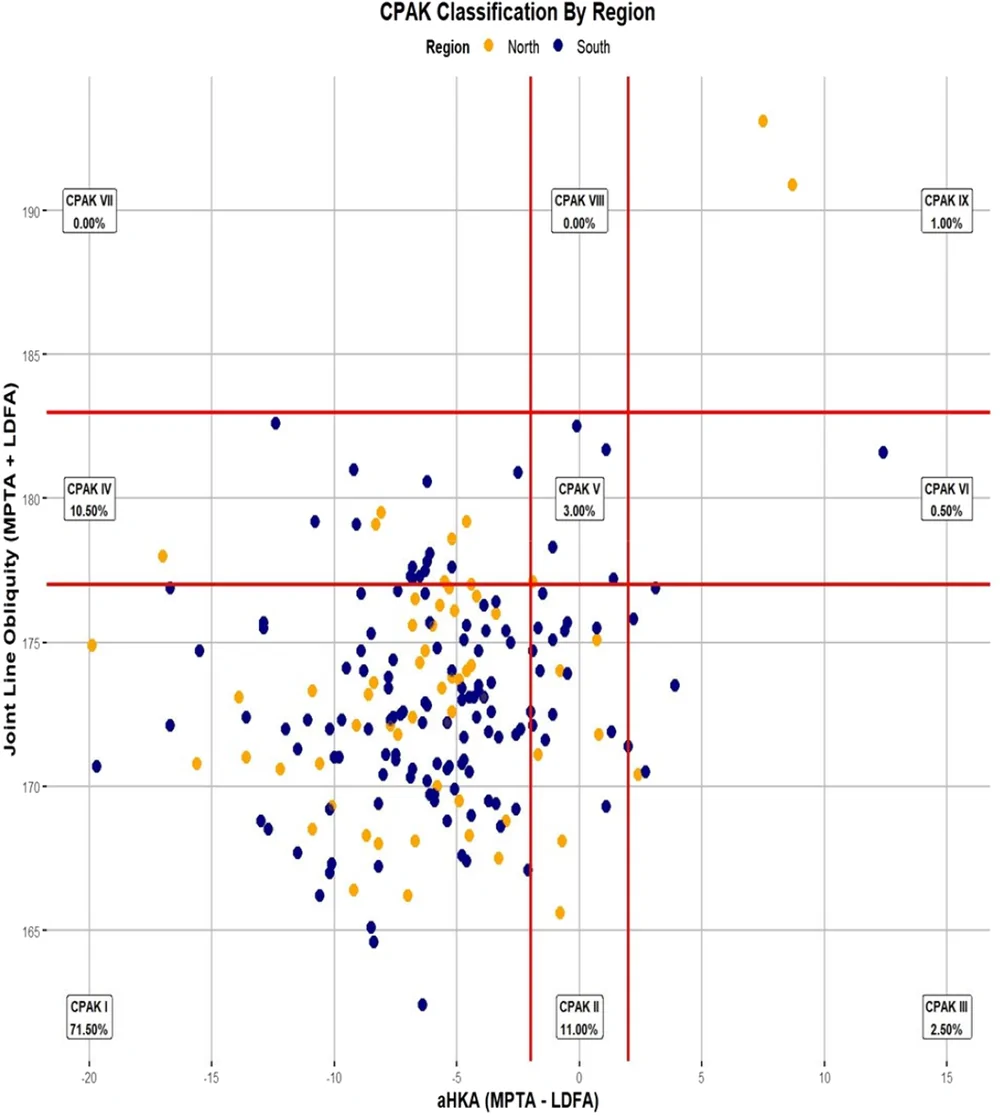
CPAK classification by region. Scatter plot of aHKA (*x*‐axis, degrees) versus JLO (*y*‐axis, degrees). Red grid lines define CPAK boundaries; type labels and percentages correspond to cohort‐level distribution. Negative aHKA values indicate varus; higher JLO values indicate a more distal joint‐line apex. This figure displays the regional distribution of CPAK phenotypes in the cohort. aHKA, arithmetic hip–knee–ankle angle; CPAK, Coronal Plane Alignment of the Knee; JLO, joint line obliquity; LDFA, lateral distal femoral angle; MPTA, medial proximal tibial angle.

Additionally, the classification distribution based on region indicated that CPAK Type I was the predominant alignment among both the northern group (*n* = 44) and the southern group (*n* = 99), demonstrating a dominant varus alignment (apex‐distal). Neutral alignment was observed in 14 cases (Type IV), 4 cases (Type V) and 1 case (Type VI) among the southern group, while the northern group only showed Type IV (*n* = 7) and Type V (*n* = 2) alignments. Detailed regional‐based CPAK classification analysis is given as scatter graph (Figure 5).

### Tailoring knee phenotype alignments

Of 71.5% of patients with a preoperative CPAK Type I phenotype, 63.4% (*n* = 127) remained Type I with aHKA values adjusted within ±3° of their baseline, while 8% (*n* = 16) shifted into Type II classification within the rKA‐defined boundaries. Type III and Type V deformity were corrected to Type IV (postoperative correction = 16%, *n* = 32). While both patients (both females) with Type IX were corrected to Type VI of rKA boundaries (Table 3). These postoperative CPAK transitions reflect the effect of set classification boundaries rather than targeted modification of phenotype. Alignment goals were based on restoring aHKA, and categorical changes occurred within acceptable rKA limits.

**Table 3:** Pre‐ and postoperative coronal alignment angles and postoperative CPAK phenotype distribution in patients undergoing RATKA using the MISSO system.

Angular values	Mean ± SD	Range (min–max)
Preoperative aHKA	174.4 ± 5.5	154–196
Postoperative aHKA	173 ± 3.2	170.3–187
Preoperative LDFA	89.51 ± 2.7	83.2–95.5
Postoperative LDFA	85.1 ± 1.2	82.9–96.2
Preoperative MPTA	83.72 ± 3.5	74.8–98.7
Postoperative MPTA	87.7 ± 1.8	84.9–94.3

CPAK‐type postoperative correction	Total, *n* (%)	
Type 1: Varus knees/apex distal (aHKA: less than minus 2 and JLO less than 177)	127 (63.5)	
Type 2: Varus knees/apex distal (aHKA 0+/−2 and JLO less than 177)	38 (19)	
Type 4: Neutral knees (aHKA less than minus 2 and JLO between 177 and 183)	32 (16)	
Type 6: Neutral knees (aHKA greater than 2 and JLO between 177 and 183)	3 (1.5)	

Abbreviations: aHKA, arithmetic hip–knee–ankle angle; CPAK, Coronal Plane Alignment of the Knee; JLO, joint line obliquity; LDFA, lateral distal femoral angle; MPTA, medial proximal tibial angle; RATKA, robotic‐assisted total knee arthroplasty; SD, standard deviation.

One patient had a tibial shaft fracture as observed during a 6‐month follow‐up duration, which was managed appropriately. No early revision surgery was noted in any of the patients.

### Functional outcomes following phenotype restoration

At 6 months, patients demonstrated satisfactory functional recovery. The mean KOOS score improved from 67.7 ± 2.1 preoperatively to 87.2 ± 1.3. KOOS daily activities scores also increased from 40.6 ± 3.5 to 80.9 ± 2.3. The FJS improved from 49.8 ± 1.3 at baseline to 75.9 ± 1.4 at 6 months (Table 4).

**Table 4:** Association between postoperative alignment adjustments (aHKA, JLO and CPAK classification) within rKA boundaries (±3° of prearthritic alignment) and functional performance.

Factors	Pain	Symptoms	Quality of life	Function	Knee injury and Osteoarthritis outcome score (Total)	Forgotten joint score
JLO adjustment	*p* < 0.05	*p* < 0.05	*p* < 0.05	*p* < 0.05	*p* < 0.05	*p* < 0.05
aHKA adjustment	*R* ^2^ = 0.05 *p* < 0.05	*p* < 0.05	*p* < 0.05	*p* < 0.05	*R* ^2^ = 0.05 *p* = 0.05	*p* < 0.05
CPAK adjustment	*p* < 0.05	*p* < 0.05	*p* < 0.05	*p* < 0.05	*p* < 0.05	*p* < 0.05
CPAK adjustment in varus patients	*p* < 0.05	*p* < 0.05	*p* < 0.05	*p* < 0.05	*p* < 0.05	*p* < 0.05
CPAK adjustment in neutral patients	*p* < 0.05	*p* < 0.05	*p* < 0.05	*p* < 0.05	*p* < 0.05	*p* < 0.05

Abbreviations: aHKA, arithmetic hip–knee–ankle angle; CPAK, Coronal Plane Alignment of the Knee; JLO, joint line obliquity; rKA, restricted kinematic alignment.

Correlation analyses identified statistically significant but weak associations between alignment changes (aHKA and JLO) and functional outcomes (*R* ^2^ ≈ 0.05; *p* < 0.05). These low coefficients indicate limited explanatory strength and suggest that early functional recovery is influenced by factors beyond coronal alignment alone.

## DISCUSSION

This study assessed the knee characteristics of individuals with arthritis undergoing RATKA using rKA with CPAK type categorization system, which was implemented in both northern and southern regions of India. The analysis determined that the patients were prone to having mostly varus knees, Type I with oblique apex distal JLO, followed by a neutral knee alignment. In this multi‐centre study, we found that the rKA approach could be applied using robotic assistance (MISSO joint system), with postoperative alignment falling within the intended aHKA limits on radiographic assessment. To the best of our knowledge, this is one of the first large‐scale reports on the rKA implementation in an Indian population using the MISSO robot. Our findings highlight several important points: (1) Precision of alignment: With RATKA, consistent alignment within rKA boundaries was achievable, with most cases within ±3° of planned alignment. (2) Phenotype preservation: Approximately two‐thirds retained their preoperative CPAK classification and shifts into adjacent categories occurred within acceptable rKA limits. These transitions reflect CPAK categorization boundaries rather than intentional modification of phenotype. (3) Early functional benefits: Patients experienced substantial improvements in pain and function by 6 months. While outcomes were favourable across all groups, knees closer to neutral alignment appeared to show numerically higher PROMs; however, these differences were not statistically significant, and our study was not powered to detect subgroup effects; therefore, they should be regarded as descriptive observations only. Further, these short‐term outcomes should be interpreted cautiously, as prior studies have shown that reproducing the patient's constitutional alignment, rather than enforcing mechanical neutrality, can achieve equal or satisfactory functional outcomes [7, 19, 26].

A study by Mulpur et al. from India investigated the distribution of CPAK phenotypes, providing a detailed comparison between healthy young individuals and patients with OA [16]. While their findings offered valuable insights, the study was limited by its single‐centre design, which restricts the generalizability of the results to the broader Indian population. Despite these limitations, their work laid an important groundwork for further research in this area. Building on this foundation, our study reports CPAK distribution data from a larger and more diverse cohort across five high‐volume arthroplasty centres located in both northern and southern regions of India. Consistent with the observations by Mulpur et al. [16], we found that the majority of arthritic patients—irrespective of age—exhibited either apex‐distal varus or neutral knee alignment patterns.

In another study reported among the Japanese population [24], the majority of arthritic patients (53.8%) exhibited Type I arthritis, characterized by varus alignment of the HKA joint and a distal location of the joint line apex. However, in the European Australian population survey [14], Type I constituted a mere 19.4%, whereas it represented a significantly higher proportion in the Japanese study. According to our observation, similar to the Japanese and Korean population [25], the majority of our Indian patients with arthritis exhibited a Type I knee phenotype (143 knees, 71.5%), characterized by a natural inward angulation of the knee and a JLO that slopes downwards towards the distal end.

A study conducted by Samant and Desai [21] examined the prevalence of several types of CPAK among healthy individuals and those with OA. The study found that Types I, II, IV and V were commonly observed in both groups. Additionally, the study found that Types VII, VIII and IX were rare overall, which aligns with the findings of MacDessi et al. [13]. The researchers determined that the CPAK method is a dependable tool for accurately predicting the natural knee structure, even in Indian patients with arthritic knees, based on their aHKA. This further substantiates the dependability of aHKA for Indian knee joints, which we agree as in our study, we found a similar trend with Type I being a dominant alignment irrespective of age, gender and region.

The findings of a retrospective multicenter study evaluating conventional TKA performed with a MA strategy showed that only 37.1% of patients achieved strict MA (corresponding to CPAK Type V), while 48.9% reached a neutral aHKA [28]. Notably, 85.4% of patients demonstrated a modification of their preoperative phenotypic alignment, and functional scores were reported to be higher in those in whom the native phenotype was restored. In contrast, in the present study, patients undergoing RATKA with a rKA strategy achieved postoperative alignment within the predefined rKA boundaries of 0° ± 3°. The postoperative CPAK distribution reflected phenotype‐informed alignment execution, with most knees either maintaining their native classification (63.4%) or transitioning into adjacent, biomechanically congruent categories (Type II: 19%, Type IV: 16% and Type VI: 1.6%). Both patients with preoperative Type IX alignment were categorized as Type VI postoperatively. These observations indicate that alignment targets were achieved within rKA limits, addressing a limitation frequently reported with conventional MA‐TKA, although this should not be interpreted as confirmation of precise resection accuracy in the absence of postoperative CT validation.

The same multicentre study [28] suggested that restoration of natural alignment parameters, including aHKA and JLO, may be associated with improved functional outcomes. While a direct causal relationship between alignment restoration and function was not established in our cohort, patients demonstrated significant improvements in KOOS and FJS scores at 6 months. However, although correlations between alignment changes and functional outcomes reached statistical significance, the coefficients of determination were low (*R* ^2^ ≈ 0.05), indicating weak associations. These findings suggest that postoperative functional recovery is influenced by multiple factors beyond coronal alignment alone. Accordingly, while alignment was maintained within rKA limits across the cohort, the observed functional improvements should be interpreted as reflecting expected postoperative recovery rather than alignment‐driven effects.

Recent work on RATKA using FA or other personalized alignment strategies provides useful context for our findings. A recent systematic review and meta‐analysis by Giurazza et al. highlighted that coronal alignment phenotypes vary significantly with geographic and demographic factors [3]. Lustig and colleagues have described FA with CT‐based robotic platforms, demonstrating that phenotype‐informed planning can be executed safely in varus and valgus morphotypes with satisfactory short‐term patient‐reported outcomes [22]. Londhe et al. demonstrated that robotic‐assisted FA leads to reproducible, patient‐specific femoral rotational positioning compared with fixed rotational references, without an associated increase in early patellofemoral complications, supporting the safety of individualized alignment execution [9]. Building on this, Diquattro et al. reported that FA‐based RATKA resulted in significant improvements in anterior compartment radiographic parameters and patient‐reported outcomes at mid‐term follow‐up, irrespective of patellar resurfacing [2]. Other series of robotic FA report good mid‐term functional results and low revision rates in large cohorts, suggesting that FA can be applied consistently across age and sex subgroups [6]. Comparative studies of robotic FA versus mechanically aligned manual TKA have shown better or similar patient‐reported outcomes and higher satisfaction at two years, reinforcing the feasibility of personalized alignment when delivered with robotic assistance [1]. In parallel, robotic platforms have also been used to compare MA and rKA, with rKA more closely restoring constitutional alignment while achieving comparable short‐term clinical outcomes [11]. Taken together, these reports indicate that modern robotic systems can support different personalized alignment philosophies (FA, KA, rKA) with acceptable safety and functional recovery. Our study adds to this literature by focusing specifically on rKA in a predominantly varus, South Asian cohort characterized using CPAK, showing that phenotype‐informed planning and limb‐level alignment within rKA limits can be achieved with satisfactory early functional outcomes. To our knowledge, no prior multicenter Indian study has combined preoperative CT‐based phenotype classification with postoperative robotic‐assisted alignment assessment, making this dataset a valuable early contribution to understanding phenotype‐informed rKA in this population.

### Implications

These results should be interpreted within the constraints of the retrospective design, single‐arm format and radiographic rather than CT‐based confirmation. Findings from this study suggest that applying rKA protocol through RATKA is feasible across diverse Indian knee phenotypes. The consistent achievement of alignment within rKA‐defined limits and the observed early functional improvement indicate that phenotype‐based surgical planning can be executed reliably using a fully automated MISSO joint robot. For surgeons, this reinforces the practicality of phenotype‐oriented planning when guided by objective robotic precision. For researchers, the data provide a baseline for future prospective and comparative studies exploring whether these early results persist long‐term and how they relate to implant survival and patient satisfaction.

### Future research

Going forward, it will be important to investigate whether these early observations persist long‐term and whether they differ meaningfully from alternative alignment strategies. Also, while our study focused on rKA, there are other personalized alignment concepts (e.g., FA, iKA) emerging. Comparative studies between these approaches, ideally randomized trials, would clarify if one strategy is optimal or if they all converge on similar outcomes when executed well. Finally, patient selection for rKA deserves attention. Our data suggests that patients with extreme varus can still benefit from rKA, but they may need a hybrid approach (partial correction). Identifying which patients should not get kinematically aligned (e.g., very large deformities or certain ligament insufficiencies) is critical.

## LIMITATIONS

Our study is limited by its retrospective, single‐arm design without a comparator group, which precludes conclusions about superiority over manual TKA or other robotic platforms. However, no patient was excluded based on adverse outcomes; notably, the case with a tibial shaft fracture at 6‐month follow‐up (managed appropriately) was included, minimizing selection bias. Although powered for functional outcomes, subgroup analyses such as by CPAK phenotype may be underpowered and should be interpreted cautiously. Postoperative CPAK transitions reflect classification boundaries rather than restoration intent. Without a comparator arm, these observations are descriptive and not inferential. Furthermore, all patients received the same implant, limiting applicability to other prosthesis designs and factors such as cost‐effectiveness and the surgical learning curve were not assessed. Postoperative CT validation of component position was not performed, as routine CT imaging is not part of standard postoperative follow‐up due to additional cost, radiation exposure and limited clinical value in patients with uneventful recovery. Alignment was instead assessed using standardized long‐leg standing radiographs, which are accepted for coronal alignment evaluation. Lastly, 6‐month results reflect early recovery; longer term follow‐up is required to evaluate the sustainability of alignment and functional outcomes. Despite these limitations, the strength of our study lies in being the first multicenter Indian analysis of CPAK phenotypes in RATKA, covering patients from diverse regions and age groups using a fully automated robotic platform (MISSO).

## CONCLUSION

This multicenter study characterized coronal knee phenotypes using preoperative CT‐derived alignment parameters and demonstrated the predominance of CPAK Type I varus configuration in an Indian cohort. RATKA (using MISSO joint robot) performed with a rKA approach achieved postoperative radiographic alignment within the planned aHKA limits across phenotypes. Early functional outcomes at 6 months showed satisfactory improvement in KOOS and FJS, consistent with expected postoperative recovery, and without early revision procedures. These findings indicate that phenotype‐informed alignment planning using robotic assistance can be executed across the spectrum of coronal morphologies encountered in this population.

## AUTHOR CONTRIBUTIONS

**Ravi Teja Rudraraju**: Conception and design of the study; acquisition; analysis and interpretation of data; drafting the article or revising it critically for important intellectual content; final approval of the version to be submitted. **Sanjay Bhalchandra Londhe, Ponnanna Karineravanda Machaiah, Supreet Bajwa, Kunal Aneja, Police Jayaram Reddy, Kanakanala Jangi Reddy** and **Dolly Singh**: Drafting the article; revising it critically for important intellectual content; final approval of the submitted version.

## CONFLICT OF INTEREST STATEMENT

Dolly Singh is a full‐time employee of Meril Healthcare Pvt. Ltd., Gujarat, India. The remaining authors declare no conflict of interest.

## ETHICS STATEMENT

The authors have nothing to report.

## Data Availability

The authors have nothing to report.
